# A multi-dimensional indicator for material and energy circularity: Proof-of-concept of exentropy in Li-ion battery recycling

**DOI:** 10.1016/j.isci.2023.108237

**Published:** 2023-10-17

**Authors:** Minerva Vierunketo, Anna Klemettinen, Markus A. Reuter, Annukka Santasalo-Aarnio, Rodrigo Serna-Guerrero

**Affiliations:** 1 Department of Chemical and Metallurgical Engineering, School of Chemical Engineering, Aalto University, P.O. Box 16200, 00076 Aalto, Finland; 2 SMS-Group GmbH, Eduard-Schloemann-Straße 4, 40237 Düsseldorf, Germany; 3 Department of Mechanical Engineering, School of Engineering, Aalto University, P.O. Box 14400, 00076 Aalto, Finland

**Keywords:** Chemistry, Electrochemical energy storage, Energy Modelling, Materials science, Materials chemistry

## Abstract

Recycling processes are an important stage in the raw material life cycle, as it enables the transition from a linear economy into a circular one. However, the currently available indicators of productivity in recycling technologies respond to the needs of a linear economy. In this work, a parameter called “exentropy” is proposed, offering the possibility to simultaneously account for mass preservation and the energy efficiency of transformative stages. As a proof-of-concept of this indicator, the analysis of a lithium-ion battery recycling process under various concentrations of a leaching reagent (i.e., 0.1M, 1M, and 2M) is presented. It is shown that, when the energy or mass dimensions are considered independently, the processes considered optimal may have conflicting characteristics. In contrast, the multi-dimensional analysis identified the process option offering the best compromise for both material and energy preservation, an aspect closer to the goals of the circular economy.

## Highlights

• A new circularity indicator called exentropy is proposed
• Exentropy accounts simultaneously for mass and energy conservation
• A proof-of-concept for the optimization of a battery recycling process is presented
• A multi-dimensional analysis is advantageous for the design of circular solutions

## Graphical abstract

**Figure:**
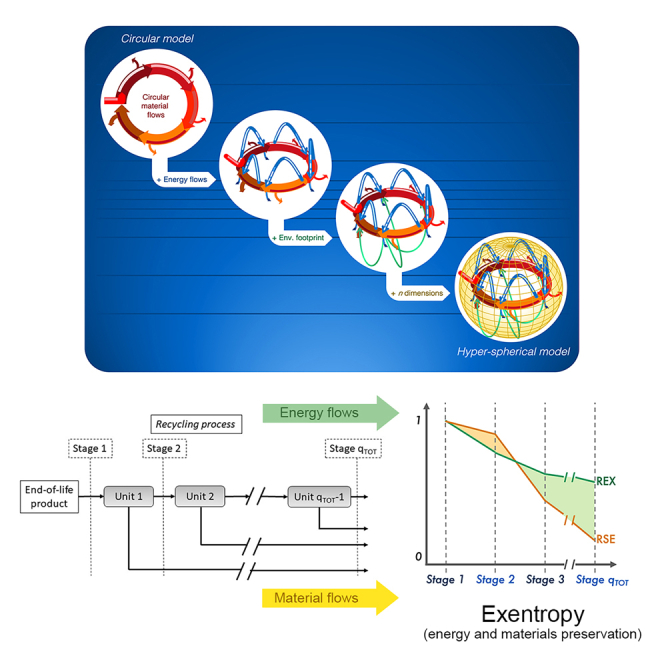

## Introduction

The expansion of human population and a global economic growth (i.e., the increasing demand of goods by the average world citizen) during the last century has substantially increased the demand of raw materials and the utilization of energy in a short period of time.^1^^,^^2^^,^^3^ This trend has inevitably lead to environmental and social problems, from the depletion of natural resources to the dependence on geographical areas with serious geopolitical conflicts.^4^^,^^5^^,^^6^ While the currently dominant linear economy model gives an erroneous assumption of unlimited resource stocks,^4^ immediate action is needed to prevent the depletion of natural resources in reality.^7^ One approach to address this challenge is that of the “circular economy” (CE), which fundamental principle is to replace the end-of-life (EOL) stage and keep value longer through the different value retention stages, such as reusing, refurbishing, or recycling.^8^ The idea of a CE model was proposed in the mid-20^th^ century, and it has gained attraction in recent years due to the risks of resource scarcity as population grows along with an increased demand of products and services.^9^ While various definitions are found in the literature,^9^^,^^10^^,^^11^^,^^12^ in general, the CE represents a regenerative system in which waste is minimized by closing, narrowing, and slowing materials and energy loops during a life cycle of a product. In this context, “closing” loops represents the recovery of materials into a value chain, “narrowing” loops refers to avoiding overly complicated routes for the circulation of materials in a useful form, while “slowing” represents the extension of the useful lifetime of a product, all of which results in a decreased consumption of resources within a given period.

Therefore, to close the loop and to transform the linear economy into circular one, EOL products are considered as resources, thus reducing the need for primary material inputs. In an ideally circular scenario, waste would not be generated at all,^4^ so the utility and value of materials would be preserved, effectively reducing the need for virgin raw materials.^5^ As the aim in CE is also to reduce energy losses in addition to material losses, circulation of energy and energy degradation should also be considered.^10^ Thus, the implementation of CE practices can further help to deal with problems indirectly associated with virgin materials production. These problems might include poor energy and materials intensity, dependency on foreign countries for critical materials, increased use of fossil energy, and even climate change.^6^^,^^9^^,^^13^ Regarding the use of energy, in CE systems the use of renewable energy sources should be prioritized to reduce emissions, and energy degradation should be studied more as we want to avoid any unnecessary energy losses.^12^^,^^14^

Despite the interest gained in the CE, there is a lack of consensus regarding the right indicators to analyze and compare technological solutions required for its implementation.^2^^,^^15^ Without indicators accounting for the CE goals by definition, there is a risk that processes aimed at recovering economically valuable materials are energy intensive, consume hazardous reagents, or produce undesired emissions. Therefore, objective, scientific, and systemic indicators of materials circularity are needed for the design and evaluation of industrial ecosystems.^8^^,^^10^ Circularity parameters are fundamental, both to support decision making and to serve as a framework for technological innovation.^1^

To shift the recycling principles toward CE, a wide variety of recyclates should be concentrated to a degree where they can be reincorporated into the value chain with minimum losses to the environment. To study the concentrating action of material processing systems, the concept of statistical entropy (SE) can be applied to describe the distribution pattern of an element or substance through a system.^16^ The concept of SE was created by Shannon and Boltzmann,^17^ who borrowed the concept of thermodynamical entropy, introduced by R. Clausius in 1865,^16^ and applied it to information theory. SE aims to represent the loss or gain of information in a system, whereas thermodynamical entropy, which is based on the 2^nd^ law of thermodynamics, represents the creation of disorder, or randomness of energy in microsystems. Even though SE and thermodynamical entropy follow a similar mathematical form, SE is measured in information bits [bit], and therefore, no physical relationship exists between the two entropy terms.^16^ Statistical entropy analysis (SEA) was proposed by Rechberger and Brunner,^16^ who combined material flow analysis (MFA) with Shannon’s SE function. According to MFA, a system consists of stages (q), transformative processes, i.e., units (u), and streams (s). A visualization of this concept can be found in supplemental information (Figure S1). The purpose of MFA is to balance all material flows in a process by systematically accounting for all input and output streams feeding and resulting from the transformative processes at every stage. The SE in each stage is determined by the concentration of each component (i) in each stream where it is present. Accordingly, an increase of SE represents the dilution of substances while the SE value of highly concentrated materials approaches to zero. Therefore, SEA can provide a systemic view of all concentration changes occurring in a system.

To compare different elements inside a system, we can use an indicator known as relative statistical entropy (RSE), which is derived from SEA. With RSE it is possible to make clearer comparisons between different elements and components in the same system, as it standardizes the entropy value of each component using their theoretical maximum as a benchmark.^18^ Furthermore, RSE can be used to calculate the substance concentrating efficiency (SCE), an indicator utilized to show the ability of a system to concentrate substances. Whereas the values of RSE goes from 1 to 0, where 1 indicates a diluted and 0 a concentrated material, the values of SCE are from 0% to 100%, where 0% indicates a diluted and 100% a concentrated material.

The aim in recycling processes is to obtain the lowest possible values of RSE, as this represents an efficient separation of the mixed materials feed.^19^ More concentrated resources are easier to control, manage, and utilize as valuable products, thus avoiding their downcycling or irreversible losses,^20^ which supports the concept of closed loop recycling. In the production stages, highly concentrated raw materials with a low entropy content are combined to create functional products. The resulting mixed substances in manufactured goods have a higher entropy, in addition to the intrinsic entropy generated by the inefficiencies of manufacturing and distribution stages. By the EOL stage, these products are randomly mixed with other products, resulting in the stage with highest entropy of the product life cycle. Without further action, this status will remain unchanged and thus, a resource efficient economy is only possible by applying efficient waste management strategies. Under this perspective, recycling processes have the aim of transforming these high-entropy mixtures into low-entropy recycled materials.^20^

While the study of material flows is fundamental for the analysis of processes from the CE perspective, energy preservation is widely acknowledged to be another relevant dimension for the analysis of materials circularity.^10^ Indeed, the recovery of valuable materials should consider the energy consumption and losses associated with each transformative process. A suitable methodology that accounts for the preservation of energy is exergy analysis (ExA). Unlike measurements of energy consumption, exergy is a property that helps quantifying the irreversible losses of useful energy.^21^^,^^22^ In the context of CE, it is important to identify whether the useful energy introduced in the system can be further utilized or whether transformative process dissipate it permanently.^21^ The ExA helps to identify and quantify the sources of exergy destruction (Ex_D_), which should be minimized for a more efficient use of resources.^23^^,^^24^ Ex_D_ can also be evaluated using an indicator called relative exergy content (REX), which is a fractional value representing the exergy content of a stage relative to the total exergy content of the system input. When exergy is being destroyed, the exergy content of a stage is being reduced. Therefore, the value of REX at the final stage can also be represented as exergy conservation efficiency (ExCE), an indicator to present how much exergy has been preserved during the process. Analogous to RSE and SCE for, the value of REX goes from 1 to 0 and values of ExCE from 0% to 100%, representing no exergy destruction at all and a completely exergy destructive system, respectively.

As indicators are needed to evaluate CE in different systems, the literature offers examples on the application of SEA to evaluate the recyclability of various materials and products, such as plastics,^25^^,^^26^^,^^27^ construction materials,^28^ electronic waste,^29^^,^^30^ thermoelectric devices,^31^ and lithium-ion batteries (LIBs).^18^^,^^32^ Furthermore, ExA has been used in the evaluation of power plants and processes and their internal recycling,^33^^,^^34^^,^^35^ performance of recycling systems in general,^36^ and recycling of specific materials and EOL products, such as coated magnesium,^37^ battery recycling systems,^22^^,^^38^^,^^39^ and the utilization of waste heat in hybrid and electric vehicles.^40^ In addition, ExA has been previously proposed as a tool to study the efficiency of energy utilization in systems involving material loops.^41^^,^^42^^,^^43^ However, no studies have tried to combine both SEA and ExA in the field of recycling, to the best of the author’s knowledge. Alternatively, SEA has been combined with energy in a recently published study by Moyaert et al.^44^ to evaluate how different types of deli packaging material affect their recycling potential. Also, SE and exergy were combined with a third parameter, entransy, in a work by Vakalis et al.^42^ to compare the efficiency of different gasification plants. Since our study will not focus on the possible utilization of heat in the process, the concept of entransy cannot be applied, as it is used to study heat transfer potential in a system.

In a previous study by our research group (Velázquez-Martínez et al.^32^), a comparison between SEA and exergy efficiency analysis was presented to showcase the limitations of the ExA when dealing with separation processes with equal energy consumption but different materials concentration efficiency. Nevertheless, the objective of such study was to demonstrate the relevance of a systemic-level analysis of materials flow, rather than the combination of parameters for materials and energy preservation. Also, both material and energy balances obtained from a simulation of a PET recycling process were used in a study by Singh et al.*,*^45^ where they conducted a comprehensive analysis of PET recycling via a simulation study. They however did not combine these two parameters, but instead used material and energy balances to obtain information for the process analysis.

**Figure 1:**
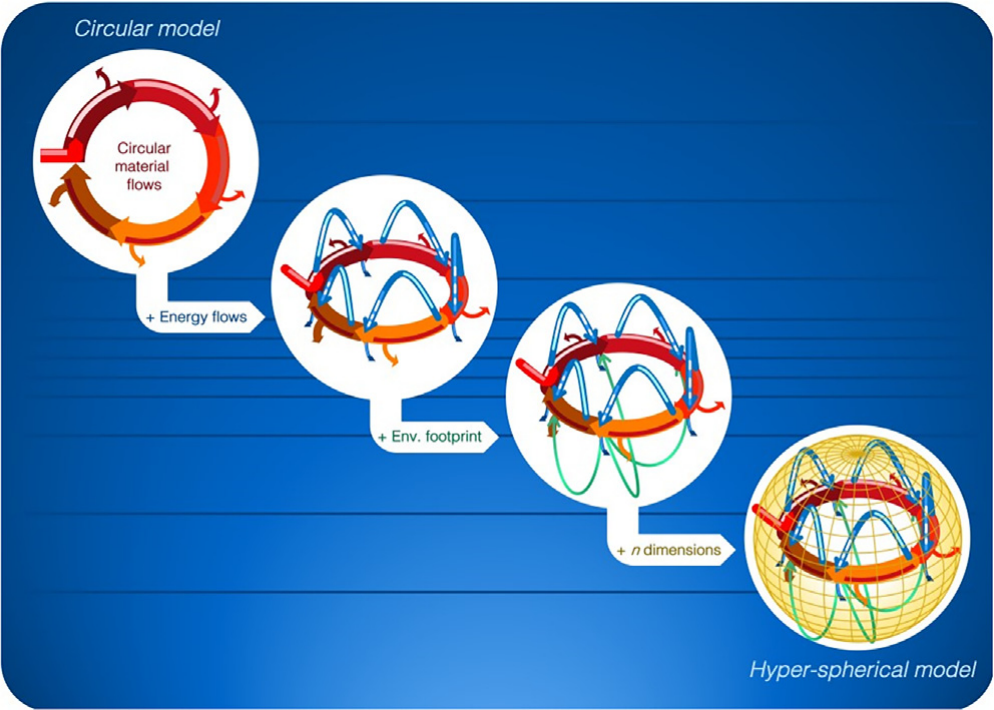
The concept of hyperspherical engineering: from circular to spherical and further to a hyperspherical solution

Evidently, indicators based solely on either material or energy balances can only capture a partial picture of the CE needs.^46^ A successful combination of the material-centric description with thermochemistry, and therefore exergy, is the key to gain a better understanding of circular systems.^24^ Exergy represents the maximum rate of work available when the system is brought into equilibrium with the surrounding system. From the perspective of raw materials, the analysis of circularity must have materials flow as basis, ideally regenerating itself with minimum losses (red arrows in Figure 1). The analysis of this circular model however should be enriched with the addition of other relevant dimensions following the definition of CE. Figure 1 shows, for example, how energy flows (represented in blue) and environmental footprints (represented in green) are intrinsically associated with the one-dimensional circularity of materials. As more dimensions of study are added in the analysis (e.g., time, quality, costs), a one-dimensional circular model will evolve into one evoking an n-dimensional hypersphere.

Therefore, the new concept introduced here is called “exentropy” (χ), which offers a system-level analysis of material and energy preservation simultaneously, thus providing a more robust evaluation of circular solutions. The principle of χ is that an irreversible loss of exergy, i.e., Ex_D_ is only justified by the effective concentration of materials. Namely, the proper way to close the material loops is to direct energy resources in a useful form toward the recovery of materials. For instance, in systems where the design of products dies not consider recyclability, the recovery of materials will result in a significant use of energy and its consequent Ex_D_. As well, suboptimal recycling processes that consume excessive energy or generate significant material losses will likely result in unjustified Ex_D_ to produce useful secondary raw materials. Thus, the concept of χ hereby proposed represented the first hyperspherical engineering indicator ever proposed, as the concept combines circular material flows with energy flows.

To showcase the newly proposed methodology, the study of a hydrometallurgical LIB recycling process, based on the Retriev process^47^^,^^48^^,^^49^^,^^50^ is hereby used as a proof-of-concept. The process was simulated using HSC Chemistry -software^51^ to obtain the stream composition and energy values needed for the calculation of RSE, REX, and exentropy. With the aim of providing a useful proof-of-concept on exentropy analysis, the process simulation was performed under various concentrations of LiOH (namely, 0.1M, 1M, and 2M) used in the Li solubilization stage. As will be further explained in the Results and discussion section, this was a carefully chosen variable since it produces conflicting efficiency values between RSE and REX by affecting both values differently in each scenario. Thus, exentropy was applied to find optimal process conditions satisfying both materials and energy conservation.

## Results and discussion

**Figure 2:**
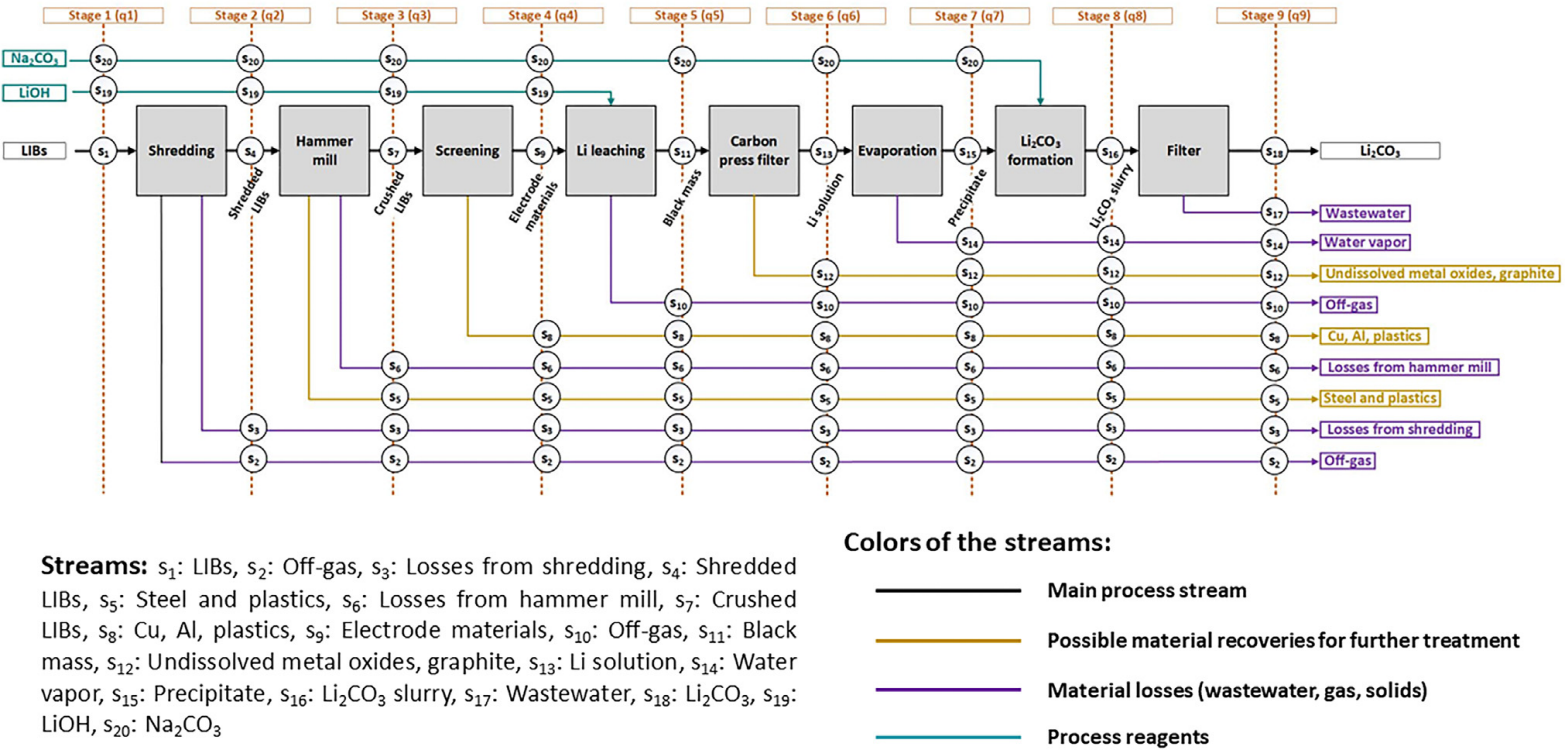
The Retriev process for LIB recycling according to MFA methodology with different streams (s), stages (q) and units (u)

For the calculation of RSE, Figure 2 was drawn to show the schematic representation of the Retriev process according to MFA methodology. The mass flows of each stream can be found in the supplemental information (Tables S1–S4).

### The values of relative statistical entropy were reduced in the system throughout all the scenarios

**Figure 3:**
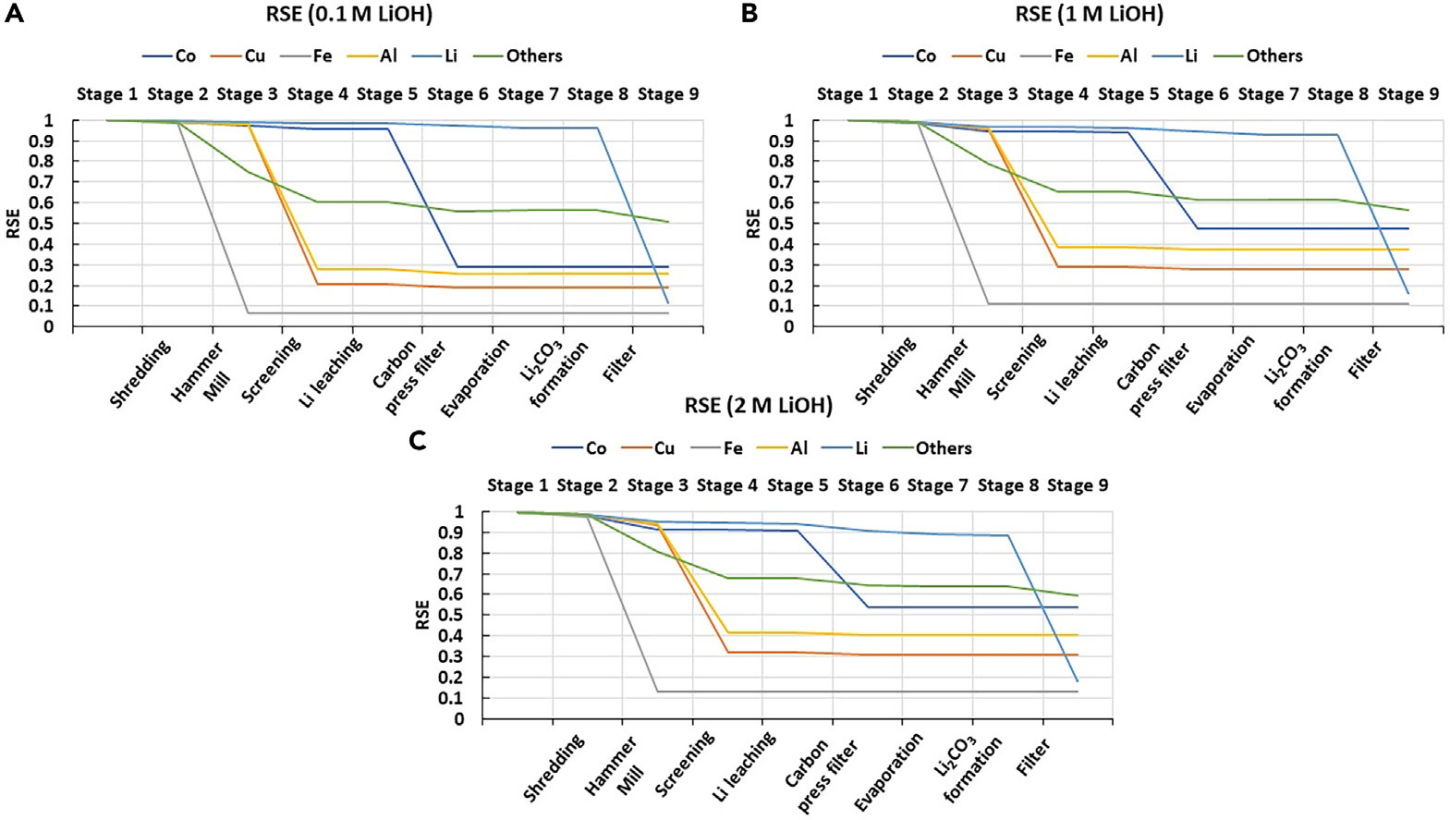
RSE of elements for different concentrations of LiOH in the Retriev process for LIB recycling: (A) 0.1M, (B) 1M, (C) 2M

The SEA results for the three different scenarios hereby explored are presented in Figure 3.

As seen, the values of RSE for all elements significantly decreased by the end of the process in every scenario. In all cases, Fe reached its lowest RSE value after the hammer mill, and the current collector materials (i.e., Al and Cu) were concentrated mainly during the screening step and slightly during carbon press filter. After leaching, Co (in the form of oxides) and graphite were separated as a mixture in the carbon press filtering step together with some remaining Al and Cu. The last change in RSE is found in the filtering step, where a concentrated Li stream in the form of Li_2_CO_3_ is obtained as a final product. The behavior of RSE of all elements is similar in each scenario, but the values of RSE increase slightly when the concentration of LiOH is increased. For example, with Cu and Al, the values of RSE are ca. 10% lower with the 0.1M LiOH scenario compared with the concentrated ones.

As mentioned earlier, the aim of recycling processes is to concentrate materials, so the values of RSE should be as close to zero as possible. As explained in “Method details” in Equation 8, a value of SCE can be calculated based on the value of RSE. Therefore, processes with a high value of SCE have a low value of RSE, as seen in Table 1, where the values of SCE were highest with 0.1M scenario.

**Table 1:** Substance concentration efficiencies (SCEs) for different elements with different concentrations of LiOH in the Retriev process

Element	SCE, 0.1M LiOH (%)	SCE, 1M LiOH (%)	SCE, 2M LiOH (%)
Co	71	52	46
Cu	81	72	69
Fe	94	89	87
Al	74	63	60
Li	75	62	56
Others	45	39	36

#### The reducing relative statistical entropy values indicate the concentration of materials

The reduction in the RSE means that all materials were concentrated by the end of the process, even when accounting for potential losses, as expected in an efficient recycling process. Because Fe reached its lowest value after hammer mill, it means that it was recovered quite efficiently from the battery scrap as most of the steel casing remains as coarse particles that are easily removed after this stage. As explained more detailed in the “Method details” section, Fe is most likely recovered after the hammer mill and prior screening, and therefore in this study, it is considered to be separated from other materials during milling. The reduction of RSE of the current collector materials occurred mainly during the screening step as it is explicitly aimed at separating these coarse particles from fine electrode materials. The reductions in the RSE values for cathode metals, i.e., Co and Li, occurred mainly during hydrometallurgical processing step, as this part of the process was mainly for treating the black mass. There was no significant reduction in the RSE value during evaporation and Li_2_CO_3_ formation, as the amount of water evaporated was relatively small compared to the mass of the mixture, and Li_2_CO_3_ formation did not intend to concentrate materials, but to form the compound “Li_2_CO_3_”, respectively.

Following the report by Bertuol et al.,^52^ it was assumed that some residues of the battery components were lost during shredding and milling due to the intrinsic separation inefficiencies of these processes. This affects the value of RSE by increasing it slightly, hence, n the elements reached an RSE value of zero. Fe was the only element considered to be in elemental form, but for the other elements, they were recovered either as mixtures (e.g., Cu and Al), or compounds (e.g., Co and Li as CoO and Li_2_CO_3_, respectively). This also affects the RSE values of the elements by increasing the values from zero. However, following the concentration efficiency as elements instead of different compounds is easier, as with pure elements only the element of interest can be followed instead of following several different compounds containing the same element.

The boundaries of the system hereby studied were set to consider process water and chemical reagents as part of the feed streams into the process. Since RSE accounts for a system-level concentration, the presence of large streams of process water will impacts its values. Indeed, the eventual separation of large streams of process water in the 0.1M LiOH scenario is computed as a successful concentration effort, resulting in a consistently lower value of RSE for all components. In reality, handling large water flows require additional efforts that are overlooked in the SEA. This exemplifies the need of additional dimensions for a robust analysis of circular systems, as will be further discussed in the subsequent Sections.

The more concentrated the materials are, the more suitable they are for production as secondary raw materials for similar applications. Therefore, to achieve a closed-loop recycling system and be more in accordance with CE, low values of RSE should be obtained at the end of a recycling process. On the contrary, in this case the value of SCE should be as high as possible, as it depends on the value of RSE in the final stage, and the lower the values of RSE are, the higher SCEs are obtained. A leakage of materials leads to material losses and therefore increased entropy in the system, thus resulting in high RSE values. Thus, RSE values should be low to obtain a more circular system. According to the results, the lowest values of RSE and therefore highest values of SCE were obtained with 0.1M scenario, so 0.1M LiOH would be the preferred option in terms of materials concentrating action.

### Exergy analysis is used to study the useful energy inside a system

**Figure 4:**
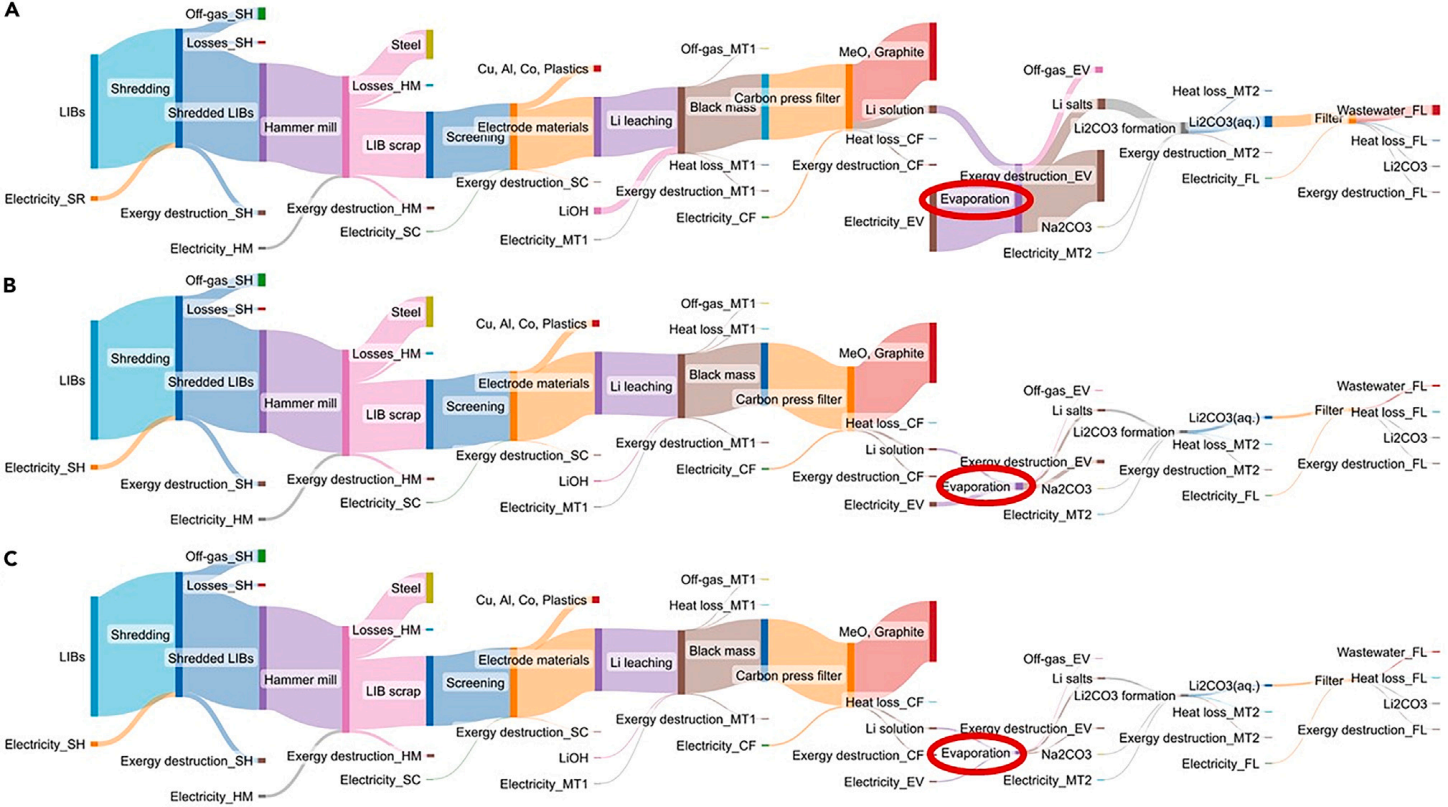
Sankey diagrams of Retriev process for LIB recycling using different concentrations of LiOH: (A). 0.1M, (B). 1M, (C). 2M

The study of exergy flows in the three systems under study are presented as Sankey diagrams in Figure 4. The Sankey diagram offers a clear visualization of all the input and output exergy streams, and the division of them into smaller streams as they are separated.^53^ The exergy values of material streams in the diagram are presented in supplemental information (Tables S1–S4).

As seen from Figure 4, the input exergy from LIBs remains generally in the process until the separation of metal oxides and graphite from the Li solution stream during carbon press filter. The biggest divisions of exergy between the input and carbon press filter occur during hammer mill and screening, as steel, Cu, and Al are recovered, respectively. After carbon press filter, the exergy streams of materials are much smaller compared to the LIB input stream, and no notable divisions of material exergy from the main stream cannot be observed. The exergy content of the final product, i.e., Li_2_CO_3_, is much smaller compared to the input exergy of LIBs.

**Figure 5:**
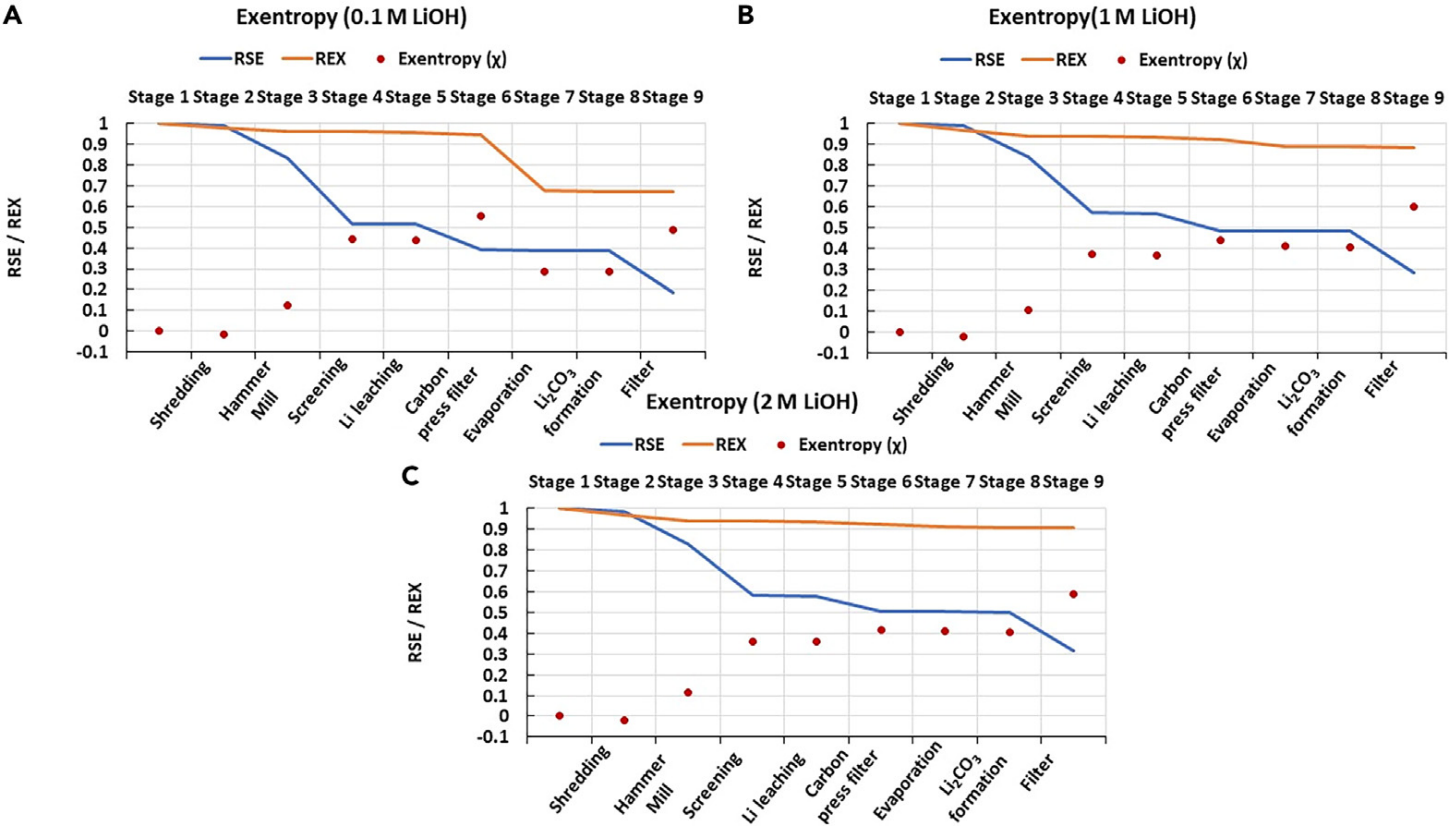
Exergy destruction, cumulative exergy destruction, and fractional exergy destruction of the Retriev process for LIB recycling using different concentrations of LiOH (A) 0.1M LiOH, (B) 1M LiOH, (C) 2M LiOH.

The other thing that can be seen in Figure 4, is that the external resource inputs, i.e., reagents and electricity, can be traced, which was not evident with RSE. Resources are needed to recover materials efficiently, which inevitably leads to the destruction of exergy.^54^ This can also be observed in Figure 4, where all units lead to some Ex_D_. The most notable change in Ex_D_ between the various scenarios occurs during the evaporation step (highlighted with a red circle) when Ex_D_ decreases while the concentration of LiOH is increased. Ex_D_ values at each stage are presented in Figure 5 to compare better Ex_D_ between each scenario.

As seen, the total Ex_D_ decreases when the concentration of LiOH is increased from 0.1M to 2M, so the scale of y axis is much smaller in 2M scenario compared to the 1M scenario. Ex_D_ can also be characterized with fractional exergy destruction (FEx_D_) curve, which visualizes more evidently the exergy distribution throughout the process. With the 0.1M scenario, there is a huge increase in FEx_D_ during evaporation, a slight increase during shredding and milling, and almost no notable increases during other units. Conversely, scenarios with 1M and 2M LiOH had a similar Ex_D_ throughout the whole process and evaporation is no longer the determinant Ex_D_ unit. In these cases, shredding and milling contributed the most to Ex_D_ due to the electricity consumption in these units.^54^^,^^55^ This can also be seen with FEx_D_, as its increase was steeper compared to the scenario with 0.1M LiOH. FEx_D_ shows also that exergy was destroyed during Li leaching and Li_2_CO_3_ formation, although this was not as significant compared with the previously mentioned stages. In conjunction, this results in ExCE values for the 0.1M, 1M, and 2M LiOH scenarios of 67%, 89%, and 90%, respectively.

#### Low exergy destruction indicates high exergy preservation in the system

The main exergy stream from LIB input to the carbon press filter is high, as this stream contains lot of elements with high elemental exergy, from which graphite has the highest molar fraction and a relatively high elemental exergy in that stream, it has the highest chemical exergy in the stream. This exergy is also much higher than the chemical exergy in the Li-containing stream, so the exergy content of the stream containing graphite is significantly higher. This is the reason also why the exergy content of the product is much smaller compared to the exergy of the input feed containing LIBs.

Because of the notable increase in FEx_D_ in the evaporation during 0.1M LiOH scenario, it clearly suggests that evaporation was the determinant Ex_D_ unit. With the other two scenarios, the steeper increase in FEx_D_ during the comminution steps indicated that comminution contributed more to Ex_D_ than with 0.1M scenario. During Li leaching and Li_2_CO_3_ formation the chemical compounds go through transformative processes, which may also cause exergy destruction together with electricity consumption.^21^ Operations such as sieving and filtering had low electricity consumption with no changes in chemical composition compared to other units, thus resulting in lower exergy destruction.

The amount of water also affects the results from the ExA. The 0.1M LiOH solution represents a comparatively large amount of water to be evaporated, associated with an extensive energy demand in comparison with the other scenarios. In exergy analysis, this is reflected by the high Ex_D_ in the evaporation unit reported for the 0.1M scenario. In addition, since the mass of water used in the process decreases at higher concentrations of LiOH, the exergy losses in the form of water vapor is comparatively minor. Thus, the inefficiencies associated with highly diluted streams become evident with ExA, unlike the SEA described above. Nevertheless, both dimensions are needed for a proper analysis, as exergy is not an indication of materials concentration.

The results showed that much of the exergy is not being destroyed, especially with 1M and 2M scenarios. This is also according to CE principles, as the aim is to have as minor exergy degradation as possible in addition to the loss of materials. As the values of Ex_D_ affects the values of REX (shown in Figure 7), Ex_D_ should be minimized to have as small energy degradation throughout the process and therefore high values of ExCE. Therefore, based on the results, the optimum process would be the scenario with 2M LiOH. This nevertheless contradicts the outcome of RSE, where the diluted LiOH was the favored scenario. A method to integrate both the analysis of mass and energy conservation is thus necessary.

### Exentropy analysis of a process combines materials concentrating action and exergy preservation

**Figure 6:**
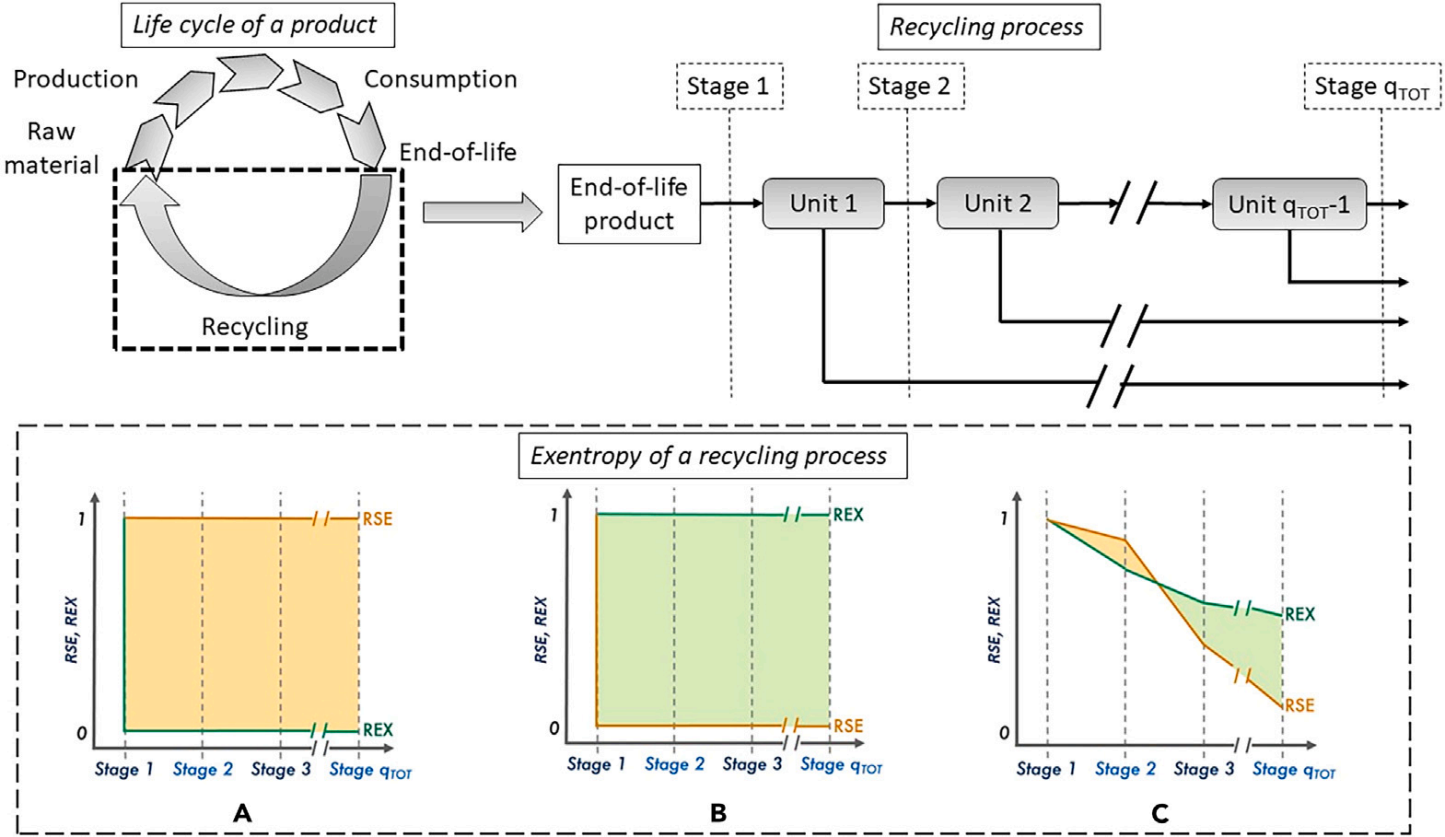
Product life cycle, recycling process according to MFA methodology, and an exentropy analysis of a process including best-case scenario: (A), worst-case scenario (B), and regular case (C)

Before going to the actual results from exentropy analysis, it is important to understand what is happening behind the concept. For the sake of clarity, the Figure 6 shows the theoretical limits and potential interpretation of χ_q_ values. For a better understanding, the figure contains the life cycle of a product and a recycling process visualized according to the MFA methodology, and three exentropy scenarios for the exentropy analysis of a recycling process. The life cycle of a product in this case consists of a production stage, i.e., processing and refining, design and manufacture, and assembly, a consumption stage, i.e., distribution, use, and EOL, and a recycling stage.^32^ As recycling is applied to recover secondary raw materials from the EOL product and thus transform the linear economy into circular one, the concept of exentropy in this article is applied to evaluate the recycling step. Exentropy evaluates the efficiency of recycling systems based on both materials concentrating action and exergy preservation, and the values are presented for each stage according to the MFA methodology visualized in Figure S1. Both RSE and REX are calculated for each stage, and the combination of these two indicators gives the values of exentropy, as seen in the lower part of the Figure 6.

In cases where the RSE curve is above the REX curve, the difference between the curves is negative, and the process can be considered inefficient, as the exergy losses outweigh the expected benefits of materials concentration. In the best-case scenario (Figure 6A), REX would remain with a value of one (i.e., no exergy losses) and RSE would be zero (i.e., production of pure streams), resulting in χ values equal to one in each stage. On the contrary, in the worst-case scenario (Figure 6B), RSE would be equal to 1 (i.e., no concentrating action occurring) and REX would be zero (i.e., all exergy is lost), and thus χ values would be −1 in each stage. In reality, processes would likely be somewhere in between, as represented in Figure 6C, in which RSE decreases as the components get concentrated but at the expense of reducing REX. If the concentrating action is however dominant (i.e., positive χ values), then the use of resources is justified, and the process can be considered efficient.

#### Exentropy requires both relative statistical entropy and relative exergy content values to be calculated

**Figure 7:**
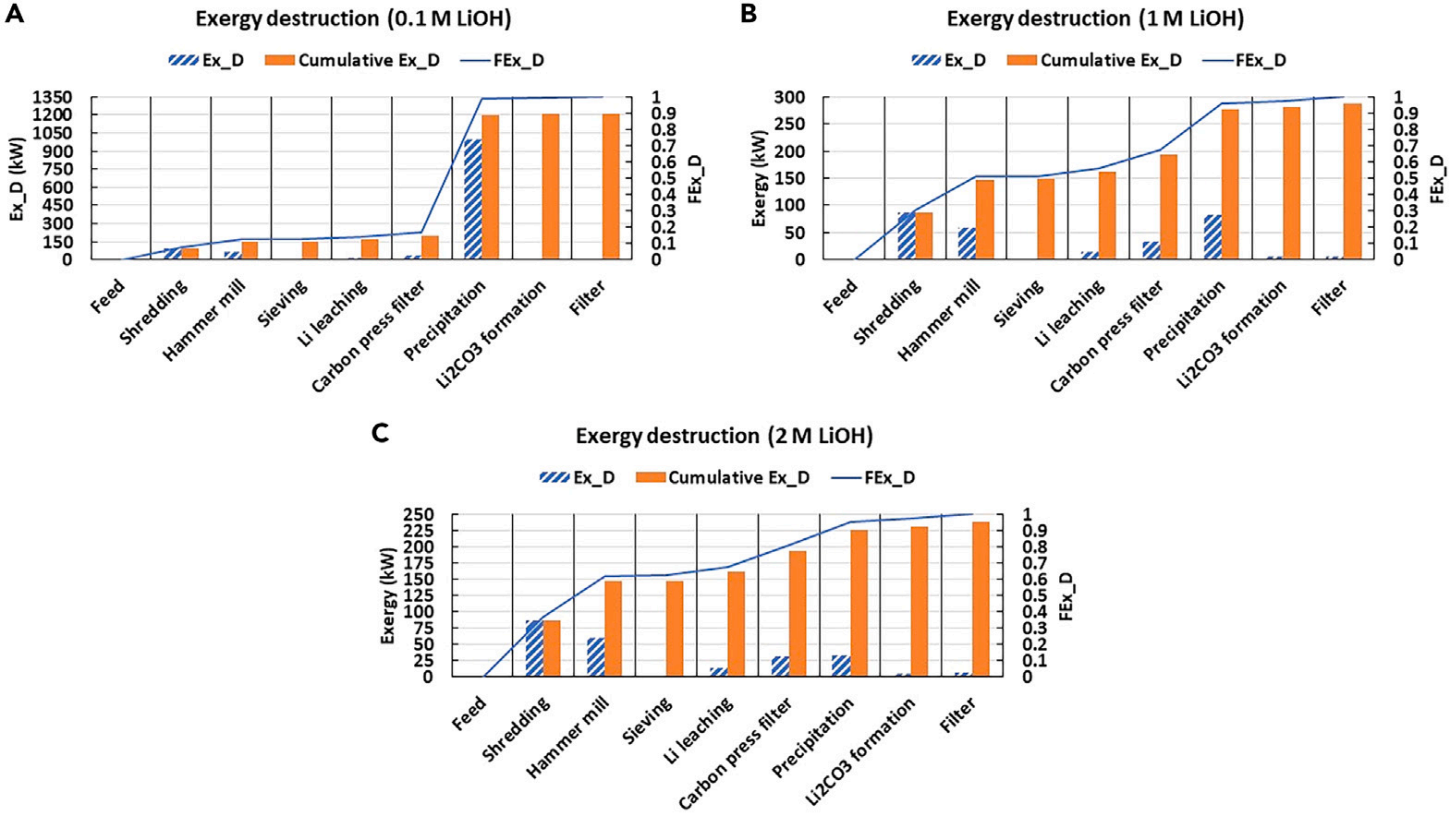
Exentropy analysis of the Retriev process for LIB recycling with different concentrations of LiOH: (A) 0.1M LiOH, (B) 1M LiOH, (C) 2M LiOH

The results from the exentropy analysis hereby proposed is visualized in Figure 7.

In the first place, it is seen that the REX values remain above those of the RSE curve during most of the process stages. The only exception is seen during shredding, where negative values of χ were obtained. After adding the χ values in all stages, ∑χ_q_ values equal to 2.75, 2.88, and 2.80 were obtained for the scenarios with 0.1, 1, and 2M LiOH, respectively. Because all scenarios had the same number of stages, there is no need to calculate the χ_eff_ values, as they will give the same conclusion than χ values alone.

#### Positive exentropy values indicate that the process is efficient in terms of material concentrating action and energy efficiency

Positive exentropy values suggest that all scenarios in this recycling process fulfill their purpose, as the materials concentration in the system (RSE) outweighs the irreversible exergy losses (REX). There is only one single stage with a negative exentropy value during the process, i.e., shredding. This is understandable since shredding is not an operation that directly results in any concentrating action, while its energy consumption inevitably leads to exergy losses. However, this is a stage required for subsequent processing, as materials liberation depends on it. Therefore, the impact of shredding on RSE reduction may become evident at a later stage of the process, illustrating the need of a system-level analysis of the process.

As seen, the methodology hereby proposed provides an overview of the system highlighting potential points for redesign and optimization. In the analysis of each individual transformative process, negative values of χ represent points where exergy destruction overshadows the benefits from the concentration of substances. Furthermore, the cumulative values of χ reflect a system-level balance of energy invested into preserving material loops, making it multi-dimensional and objective parameter to define the efficiency of processes following the CE principles. The concept of exentropy offers a novel type of analysis that could further support other parameters associated with SE and exergy, such as the aforementioned methods by Nimmegeers and Billen,^25^ Fernandes et al.*,*^41^ and Vakalis et al*.*^42^

Based on RSE and REX, both are interesting methods to analyze the efficiency of materials concentration and energy conservation in a system, respectively. Nevertheless, these are independent parameters that only evaluate a single dimension relevant for circularity. To provide a more robust analysis, a new parameter hereby named “exentropy” was proposed to evaluate both material and energy circularity simultaneously. Since a high value of REX and a low value of RSE indicate an optimal system for exergy preservation and materials concentrating action, respectively, a higher χ value represents an optimal process for materials and energy preservation. Therefore, the results suggest that it is in fact the scenario using 1M solution that is optimal, as it obtained the highest χ values. Also, as low RSE and high REX values represent a circular system, the higher the χ value is, the more circular the system is in terms of both materials and energy. This makes the method is suitable for evaluating which process is the most appropriate for closed-loop recycling, and whether the secondary materials of sufficient quality obtained could be used again in manufacturing or similar applications.

### Conclusions

A proper analysis of the circularity of process should consider the impact of transformative stages on various dimensions such as materials and energy preservation. As shown in this work, processes can be evaluated either on a material-basis using RSE or on an energy-basis using ExA, but there might be limitations when analyzing these two dimensions independently. Using these methods, it is difficult to objectively assess whether it is preferable to obtain purer material streams at the expense of higher exergy destruction or if it is better to obtain less pure streams but with a higher exergy preservation.

To address the limitation of the current methodologies, a new method called exentropy was introduced to simultaneously account for both dimensions of material concentration and energy preservation. To provide a proof-of-concept of this indicator, a recycling process was studied under various concentrations of LiOH used as an extractive reagent. The results from SEA and ExA on the recycling process case study offered different conclusions for the optimal concentration of LiOH to be used. Accordingly, it was possible to obtain low values of RSE with a high cost of exergy destruction and vice versa. In this manner, it was illustrated how χ can be used to identify the process conditions offering the optimal trade-off between materials concentration and energy preservation. Admittedly, true process optimization would require a larger number of scenarios, but this was considered beyond the aim of this proof-of-concept study.

Exentropy can be used as a tool for the evaluation and optimization of processes by identifying transformation stages with unacceptably high exergy destruction. For instance, mechanical treatment methods such as shredding will likely result in a negative contribution to the system-level exentropy as they do not directly contribute to the concentration of species. Consequently, the purpose of such unit operations needs to be justified by their impact on χ values at later stages of the system. Similarly, exentropy may also be suitable to compare different processes using a robust parameter that favors the efficient use of energy for materials recovery. This proof-of-concept represents only the first effort to provide an optimization method that accounts for the multi-dimensional nature of CE, offering the possibility to identify the most efficient process in terms of both material enrichment and energy conservation.

#### Outlook

In future studies, some limitations and assumptions made in this article must be reconsidered when approaching more realistic simulations. To have as detailed recycling processes as possible, the possibility of mixed battery chemistries in the feed should be considered, in line with the expectations in industrial recycling processes. Also, the process hereby presented considered only the battery materials flow excluding further treatment of side streams, such as off-gases. Furthermore, to have more accurate exergy calculations, mixing entropy could be considered in the calculations as well. To further expand on the analysis of a circular system, parameters covering other dimensions such as the environmental impact, and their economic implications should be considered.

### Limitations of the study

The subsequent question is whether the result from the exentropy analysis in this article is a reasonable outcome, considering it once again conflicts with the independent suggestions from SEA and ExA. Upon critical analysis, it is firstly seen that the highest decrease in RSE was indeed obtained with 0.1M LiOH, but this scenario is burdened by a significant exergy destruction in the evaporation step. Admittedly, the simulation was carried out under the assumption that LiOH concentration did not significantly affect the productivity of the system or the quality of the products. Nevertheless, as a proof-of-concept, it helps exemplify that SEA can be favored under ideal circumstances that may overlook real implications in terms of energy consumption. The opposite could be argued with respect to the favorable exergy preservation with the 2M LiOH scenario. Certainly, the most efficient way to preserve energy is to prevent its utilization. Nevertheless, the extent of transformations needed to reintroduce materials to the value chain may not be achieved by such a stationary system.

## STAR★Methods

### Key resources table

**Table:** 

REAGENT or RESOURCE	SOURCE	IDENTIFIER
**Software and algorithms**		

HSC Chemistry 10®	Metso Outotec^51^	https://www.mogroup.com/portfolio/hsc-chemistry/

### Resource availability

#### Lead contact

Further information and request for resources should be directed to and will be fulfilled by the lead contact, Rodrigo Serna-Guerrero (rodrigo.serna@aalto.fi).

#### Materials availability

This study did not generate new unique reagents.

### Method details

#### Statistical entropy analysis (SEA)

For the reader interested in the SEA-MFA methodology, it is advised to consult the seminal work from Rechberger and Brunner,^16^ and from Velázquez-Martinez et al.^32^ where it is described in further detail. A visualization of the MFA method can be found in supplemental information, Figure S1. Briefly, to calculate the SE of a component, its concentration and standardized mass fraction are needed, according to Equation 1.

(Equation 1) $$h_{i,s}=-{\dot{m}}_{i,s}c_{i,s}\;\log_{2}\left(c_{i,s}\right)\geq 0$$

where $h_{i,s}$ is SE of i^th^ component in s^th^ stream in information bits [bit], $c_{i,s}$ is the concentration of the i^th^ component in the s^th^ stream in fractional composition, and ${\dot{m}}_{i,s}$ is the standardized mass fraction of the i^th^ component in the s^th^ stream in dimensionless unit. To calculate the standardized mass fraction of a component, Equations 2 and 3 are used.

(Equation 2) $${\dot{m}}_{i,s}=\frac{{\dot{m}}_{s}}{\sum_{s}\dot{X_{i}}}$$

(Equation 3) $${\dot{X}}_{i}=\dot{m_{s}}c_{i,s}$$

where ${\dot{m}}_{s}$ is the mass flow of the s^th^ stream, $X_{i}$ is the total substance flow of component i in the units of mass flow, and $c_{i,s}$ is the concentration of the component i in stream s in fractional composition.

After calculating SE using Equations 1, 2 and 3, it is possible to calculate the total SE in a stage, by adding the statistical entropies of all its corresponding streams as described in Equation 4.

(Equation 4) $$H_{i,q}=\sum_{q}h_{i,s}$$

where $H_{i,q}$ is the SE of the i^th^ component in the q^th^ stage. With the aim of simplifying the comparison of entropic values between different elements or components, a benchmark value can be used to calculate RSE.^23^

Typically, it is calculated using the stage of maximum entropy of in the system (H^max^) as a reference value. In the present work, the SE of the feed was used as the reference state to calculate RSE following the Equations 5 and 6:

(Equation 5) $$H_{i,q}^{\max}=H_{i,1}$$

(Equation 6) $$RSE_{i,q}=\frac{H_{i,q}}{H_{i,q}^{\max}}$$

The value of RSE is dimensionless and allows for an easy interpretation of the entropic behavior of materials flowing in a system. It is also possible to describe the collective entropy of all components in the system with Equation 7.

(Equation 7) $${RSE}_{q}^{total}=\frac{\sum_{i=1}^{k}H_{i,q}}{\sum_{i=1}^{k}H_{i,q}^{\max}}$$

where ${\text{RSE}}_{q}^{\text{total}}$ is the total RSE in the process at the q^th^ stage.

The substance concentrating efficiency (SCE)^16^^,^^56^ is calculated from the difference between input and output RSE values in a system, as described in Equation 8.

(Equation 8) $$SCE=\frac{RSE_{i,input}-RSE_{i,output}}{RSE_{i,input}}*100\%$$

Where $\text{RS}E_{i,\text{input}}$ and $\text{RS}E_{i,\text{output}}$ are the input and output values of RSE of component i in the system, respectively.

#### Exergy analysis (ExA)

The total exergy of a system consists of its physical, chemical, kinetic, and potential exergy. Kinetic exergy is a result of the velocity with respect to system boundaries, and potential exergy is given by the velocity under any given body force field.^57^ However, kinetic and potential exergy are usually neglected since they are an order of magnitude lower compared to the contribution by chemical and physical exergy.^58^ In simple terms, physical exergy describes the maximum work obtained, when there is a difference in temperature or pressure between the system and the environment.^54^^,^^59^ Chemical exergy describes the exergy, or usable energy, intrinsically contained in an element or compound.^60^ The system-level exergy is the addition of exergy contributed by all elements and compounds within the system boundaries. The total exergy of a system thus depends on its composition, and it may change as its components undergo transformation processes.

The total exergy of a stream can be presented as a sum of chemical and physical exergy of the components in that stream, as shown in Equation 9.

(Equation 9) $$Ex_{tot,s}\approx {Ex}_{ch,i}+{Ex}_{ph,i}$$

The process simulation software used in this work, i.e., HSC Chemistry® calculates the total exergy for species using Equation 10:^51^

(Equation 10) $$Ex_{tot}=\sum_{k}n_{k}b_{k}^{ref}+\Delta G_{f\left(25\;{}^{\circ}C,1\text{bar}\right)}^{0}+(N_{i}-N_{i\left(25\;{}^{\circ}C,1\text{bar}\right)}-T_{25{}^{\circ}C}\left(S_{i}-S_{i\left(25{}^{\circ}C,1\;\text{bar}\right)}\right)$$

where n_k_ is the stoichiometric amount of element k in the species, $b_{k}^{\text{ref}}$ is the elemental exergy of element k, $\Delta G_{f\left(25\;{}^{\circ}C,1\text{bar}\right)}^{0}$ is the standard formation of Gibbs energy of the species, N_i_ is the total enthalpy of the species at the temperature of T, $N_{i\left(25\;{}^{\circ}C,1\text{bar}\right)}$ is the total enthalpy of the species in the standard state, S_i_ is the total entropy of the species at a given temperature T, and $S_{i\left(25\;{}^{\circ}C,1\text{bar}\right)}$ is the total entropy of the species in the standard state.

Standard conditions in the calculations are usually 25°C and 1 bar, and the exergy calculations are based on Szargut theory of exergy. Solid and liquid streams are calculated in the standard pressure, and gaseous streams adds the possible pressure differing from the standard state to the exergy. In the software, elemental exergy is accounted as a part of the chemical exergy, and all values for each species are calculated separately and then added up to the total values of the streams. All the information for the calculations in this work was obtained from the HSC Chemistry®, except for temperature, pressure, number of species and their masses in the stream. A challenge with exergy calculations in HSC is that the mixing entropy cannot be associated with specific species in a meaningful way, so entropy increase due to mixing of different materials is left out from the equation. Thus, in the calculations presented in this manuscript, only the stream exergy is accounted for.

The system may also use electricity or heat in the process. In HSC®, electricity is calculated directly into the total exergy. For the heat input and losses, the software uses the Carnot cycle to convert heat to exergy, as presented in Equation 11.

(Equation 11) $$Ex_{heat}=q*\left(1-\frac{T_{0}}{T}\right)$$

where q is heat flow, T_0_ is the temperature in the standard state, and T is the temperature of the heat source.

Unlike energy, exergy does not satisfy the law of conservation. Thus, not all the input exergy is transferred from feed to products.^60^ The left-hand side of the exergy balance in Equation 12 considers the input exergy as the result of both the input exergy contained in the feed materials (Ex_feed_) and the exergy provided by external energy inputs (Ex_energy_). The terms related to the exergy output include product streams (Ex_products_), waste streams (Ex_waste_), the irreversible exergy destruction (Ex_D_), and the exergy lost in the form of heat (Ex_Q_). It is worth pointing out that the waste stream is traditionally defined as materials without economic interest, although this might not entirely follow the aims of CE where preservation of all materials is pursued.

(Equation 12) $$Ex_{feed}+Ex_{energy}=Ex_{products}+Ex_{waste}+Ex_{D}+Ex_{Q}$$

In an ideal irreversible process, Ex_D_ can be zero, meaning that exergy input equals exergy output. In reality, all stages destroy exergy due to the intrinsic inefficiencies of energy and mass transfer. The cumulative Ex_D_ shows the evolution of exergy losses as the process progresses, which may aid on identifying which units (u) are the most inefficient in terms of energy in Equation 13:

(Equation 13) $$Ex_{D,u}=\sum_{1}^{u}Ex_{D}$$

Where $Ex_{D,u}$ is the cumulative exergy destruction during each unit.

To facilitate the comparison between processes, a fractional exergy destruction (FEx_D_) can be calculated using the value of Ex_D,u_ at the end of the process (i.e., the total Ex_D_) as a baseline according to Equation 14.

(Equation 14) $$FEx_{D}=\frac{Ex_{D,u}}{Ex_{D,tot}}$$

A different approach to trace the exergy conservation is by tracing the decrease of exergy levels resulting from process inefficiencies. Every stage contains a given amount of exergy, which can be calculated by summing up the exergies in all its corresponding streams (Equation 15):

(Equation 15) $$Ex_{q}=\sum Ex_{s,q}$$

Where $Ex_{q}$ is the exergy content of the stage q and ${\text{Ex}}_{s,q}$ is the exergy content of the stream s in stage q. Evidently, the feed stage is the one with the maximum exergy content (Ex_max_) in the process, so it can be used as a benchmark value to define a dimensionless value of relative exergy (REX) as shown in Equation 16.

(Equation 16) $$REX=\frac{Ex_{q}}{Ex_{\max}}$$

The value of REX at the final stage can also be used to represent the exergy conservation efficiency (ExCE) of the process, as SCE presented the substance concentration efficiency in Equation 8.

#### Exentropy analysis

As discussed in the preceding subsections, both RSE and ExA are interesting methods to analyze the efficiency of materials concentration and energy conservation in a system, respectively. Nevertheless, these are independent parameters that only evaluate a single dimension relevant for circularity. To provide a more robust analysis, a new parameter hereby named “exentropy” is proposed.

Ideally, REX should remain as high as possible, while RSE should tend to zero. A simple way to establish the balance of such resource requirements is by setting a normalized difference between exergy and SE, hereby referred to as χ, Equation 17.

(Equation 17) $$\chi_{q}=REX_{q}-RSE_{q}$$

Where $\chi_{q}$ is the exentropy at any given state. As both REX and RSE are dimensionless values, χ is also dimensionless.

A mean to compare processes with different number of stages is the use of a normalized exentropic efficiency (χ_eff_), based on the fact that the maximum possible value of χ is the same as the total number of stages in a system, i.e., $\chi_{\max}=q_{\text{Tot}.}*1$, as in Equation 18:

(Equation 18) $$\chi_{eff}=\frac{\sum \chi_{q}}{q_{\text{Tot}.}*1}*100\%$$

Where $\sum \chi_{q}$ is the total exentropy.

#### Process simulation of a battery recycling process

A battery recycling process was chosen as a case study to test the methodology hereby proposed. Specifically, the Retriev process, formerly known as the Toxco process, was selected as it is one of the most developed battery recycling processes based on hydrometallurgical extraction with a reported capacity of 4500 metric ton of batteries per year.^47^^,^^61^

Recycling of batteries was chosen as a case study since battery raw materials are considered critical due to the ambitious electrification targets currently set for the transportation industry as some countries want to end sales of internal combustion engine vehicles and increase the share of electric vehicles in the market.^62^^,^^63^^,^^64^ In addition, LIBs are widely found in portable electronics and in energy stations supporting clean power generation. The average lifespan of LIBs varies between 2—15 years, depending on the application where they are used in.^65^^,^^66^ A lot of LIBs have already become waste, as they have been in use for 30 years, and due to the increasing production volume, there will also be a considerable growth in EOL LIBs in the next decade.^65^^,^^67^ Indeed, the number of EOL LIBs has been estimated to be 11 million metric tons in 2030.^66^ This is why better recycling solutions for batteries will be needed urgently,^68^ and therefore, different recycling processes must be evaluated using different circularity indicators.

In this study, the feed in the simulation was assumed to be 534 kg/h with a composition presented in supplemental information (Figure S2). Size fractions and elemental distributions of sieved LIB materials reported by Zhang et al.^69^ were used to estimate the distribution of graphite, LCO, Cu, and Al. The rest of the battery materials were assumed to have evenly distributed composition in each size fraction. The mechanical processing part was assumed to occur in room temperature. According to the original patent, a wide variety of techniques can be applied to the process, and several adjustments can be made without parting from the scope of the invention, which is the recovery of Li.^70^ By using the studies by Pinegar and Smith,^48^ Sonoc et al.*,*^49^ Dunn et al.^50^ and Velázquez-Martínez et al.,^47^ a process was simulated containing the stages presented schematically in Figure 3. The process was simulated with HSC Chemistry® 10 software^51^ to obtain the mass and energy balances, and to calculate the exergy content of each stream. Specifically, the process includes the following stages.

1. *Shredding:* The batteries are first shredded into smaller pieces. Shredding step produces also off-gas, which in this case is assumed to be the volatile solvents from the electrolyte. The gases are treated before their release to the environment, but this step is out of the scope of this study. It was also assumed that 2.6 wt.% of solid battery materials were lost during shredding as a result of materials remaining in the machine according to the study by Bertuol et al*.*^52^
2. *Hammer mill:* Shredded LIBs are crushed in a hammer mill, from where steel and plastics can be removed by a screen or another subsequent separating processes.^49^^,^^50^ Losses in this step was also assumed to be the same than in shredding, i.e., 2.6 wt.% of solids according to Bertuol et al*.*^52^
3. *Screening:* LIB scrap coming from hammer mill is screened with a 710 μm screen, from where coarser particles (Cu and Al) are separated from the finer particles (electrode materials). According to the simulation, recoveries of LCO, graphite, Al, and Cu into the underflow were 88.3%, 92.1%, 3.2%, and 2.6%, respectively.
4. *Li leaching:* The underflow product of the screen is treated with an aqueous solution of LiOH to extract Li from the cathode particles. This results in a Li-rich solution and an undissolved solid mixture of metal oxides and graphite. The Li-rich solution contains dissolved Li salts, whose formation depends on the battery chemistry. In this study, it was assumed that LiOH, LiF and Li_3_PO_4_ were the salts being formed. Heat balance of the unit was set to equilibrium (i.e., 0) by changing the temperature of the unit in the simulation.
5. *Carbon press filter:* The Li-rich solution formed in the previous step is filtered from the suspended solids. It is assumed that the moisture content of the cake is 28 % after the filtering step,^70^ and the Li-rich solution does not contain any of the graphite or metal oxides.
6. *Evaporation and storage tank array:* The Li filtrate is fed into a storage tank array, where it is dewatered. As the liquid is modeled to be a diluted aqueous solution, it is assumed that the liquid is heated to 100°C, and 10% of the water is evaporated. During evaporation, the concentration of Li salts in solution increases until the solution becomes supersaturated and the salts precipitate.
7. *Formation of Li*_*2*_*CO*_*3*_*:* The precipitate is reacted with soda ash (Na_2_CO_3_) in a stirred tank to form Li_2_CO_3_. Heat balance of this unit was set to zero by changing the temperature, which remained high. This is reasonable, because the formation of Li_2_CO_3_ is faster at higher temperatures^71^ and the yield is higher.^72^ Also, further dissolution of the Li_2_CO_3_ product is prevented at higher temperatures while dissolving possible Li- and Na-salt byproducts.
8. *Filter:* The Li_2_CO_3_ slurry proceeds to a filter press, where excess water is removed, and a dewatered product is obtained that can be used as a precursor reagent in the manufacturing of LIB cathode materials.^47^^,^^48^^,^^49^^,^^50^ It is assumed, that the water content of the cake after filtering is 28%.^70^

It should be noted that some descriptions of the Retriev process claim that batteries are discharged and deactivated before shredding using either brine solution or liquid nitrogen for a safer handling.^47^^,^^70^ However, there are no specific details in the publicly available literature on these discharging steps that could be used to estimate exergy flows. Since understanding this specific stage was not the main scope of this study, it was neglected from the process simulation.

The energy consumption of each unit operation and unit processes needed in the simulation were estimated from the publicly available literature and are summarized in supplemental information in Table S5. Data for energy consumption in the evaporation tank and temperatures in the hydrometallurgical processing units other than evaporation were calculated by the simulation software. All energy input were assumed to be electricity from an unknown source, and the amounts of chemical reagents used were determined by stoichiometry.

Process outline is presented in supplemental information in Figure S3.

## Data Availability

• All data reported in this paper will be shared by the lead contact upon request. • This paper does not report original code. • Any additional information required to reanalyze the data reported in this paper is available from the lead contact upon request.

## Supplemental information

Document S1. Figures S1‒S3 and Tables S1‒S5

## References

[1] Sciubba E. (2019). Exergy-based ecological indicators: From Thermo-Economics to cumulative exergy consumption to Thermo-Ecological Cost and Extended Exergy Accounting. Energy.

[2] Lucia U., Fino D., Grisolia G. (2022). A thermoeconomic indicator for the sustainable development with social considerations: A thermoeconomy for sustainable society. Environ. Dev. Sustain..

[3] Lucas R.E., Federal Reserve Bank of Mineapolis The Industrial Revolution: Past and Future. https://www.minneapolisfed.org/article/2004/the-industrial-revolution-past-and-future.

[4] Busu C., Busu M. (2018). Modeling the circular economy processes at the EU level using an evaluation algorithm based on shannon entropy. Processes.

[5] Panchal R., Singh A., Diwan H. (2021). Does circular economy performance lead to sustainable development? – A systematic literature review. J. Environ. Manage..

[6] Tomić T., Schneider D.R. (2018). The role of energy from waste in circular economy and closing the loop concept – Energy analysis approach. Renew. Sustain. Energy Rev..

[7] Araujo Galvão G.D., De Nadae J., Clemente D.H., Chinen G., De Carvalho M.M. (2018). Circular Economy: Overview of Barriers. Procedia CIRP.

[8] Garcia-Saravia Ortiz-de-Montellano C., van der Meer Y. (2022). A Theoretical Framework for Circular Processes and Circular Impacts Through a Comprehensive Review of Indicators. Glob. J. Flex. Syst. Manag..

[9] Cisternas L.A., Ordóñez R., Jeldres R.I., Serna-Guerrero R. (2022). Toward the Implementation of Circular Economy Strategies: An Overview of the Current Situation in Mineral Processing. Miner. Process. Extr. Metall. Rev..

[10] Geissdoerfer M., Savaget P., Bocken N.M., Hultink E.J. (2017). The Circular Economy – A new sustainability paradigm?. J. Clean. Prod..

[11] Bocken N.M.P., de Pauw I., Bakker C., van der Grinten B. (2016). Product design and business model strategies for a circular economy. J. Ind. Prod. Eng..

[12] Ellen MacArthur Foundation It’s Time for a Circular Economy. https://ellenmacarthurfoundation.org/.

[13] Rinne M., Elomaa H., Porvali A., Lundström M. (2021). Simulation-based life cycle assessment for hydrometallurgical recycling of mixed LIB and NiMH waste. Resour. Conserv. Recycl..

[14] Kirchherr J., Reike D., Hekkert M. (2017). Conceptualizing the circular economy: An analysis of 114 definitions. Resour. Conserv. Recycl..

[15] De Pascale A., Arbolino R., Szopik-Depczyńska K., Limosani M., Ioppolo G. (2021). A systematic review for measuring circular economy: The 61 indicators. J. Clean. Prod..

[16] Rechberger H., Brunner P.H. (2002). A new, entropy based method to support waste and resource management decisions. Environ. Sci. Technol..

[17] Shannon C.E. (1948). A mathematical theory of communication. Bell Syst. Tech. J..

[18] Velázquez-Martinez O., Porvali A., van den Boogaart K.G., Santasalo-Aarnio A., Lunström M., Reuter M., Serna-Guerrero R. (2019). On the use of statistical entropy analysis as assessment parameter for the comparison of lithium-ion battery recycling processes. Batteries.

[19] Parchomenko A., Nelen D., Gillabel J., Vrancken K.C., Rechberger H. (2021). Resource effectiveness of the European automotive sector – a statistical entropy analysis over time. Resour. Conserv. Recycl..

[20] Laner D., Zoboli O., Rechberger H. (2017). Statistical entropy analysis to evaluate resource efficiency: Phosphorus use in Austria. Ecol. Indic..

[21] Carneiro M.L.N., Gomes M.S.P. (2019). Energy, exergy, environmental and economic analysis of hybrid waste-to-energy plants. Energy Convers. Manag..

[22] Olapiriyakul S., Caudill R.J. (2009). Thermodynamic analysis to assess the environmental impact of end-of-life recovery processing for nanotechnology products. Environ. Sci. Technol..

[23] Bai L., Qiao Q., Li Y., Wan S., Xie M., Chai F. (2015). Statistical entropy analysis of substance flows in a lead smelting process. Resour. Conserv. Recycl..

[24] Reuter M.A., van Schaick A., Gutzmer J., Bartie N., Abadías-Llamas A. (2019). Challenges of the Circular Economy: A Material, Metallurgical, and Product Design Perspective. Annu. Rev. Mater. Res..

[25] Nimmegeers P., Billen P. (2021). Quantifying the Separation Complexity of Mixed Plastic Waste Streams with Statistical Entropy: A Plastic Packaging Waste Case Study in Belgium. ACS Sustain. Chem. Eng..

[26] Nimmegeers P., Parchomenko A., De Meulenaere P., D’hooge D.R., Van Steenberge P.H.M., Rechberger H., Billen P. (2021). Extending multilevel statistical entropy analysis towards plastic recyclability prediction. Sustain. Times.

[27] Liu Y., Zheng Z., Zhao L., Wang Z. (2021). Quality assessment of post-consumer plastic bottles with joint entropy method: A case study in Beijing, China. Resour. Conserv. Recycl..

[28] Roithner C., Cencic O., Honic M., Rechberger H. (2022). Recyclability assessment at the building design stage based on statistical entropy: A case study on timber and concrete building. Resour. Conserv. Recycl..

[29] Zeng X., Li J. (2016).

[30] Roithner C., Cencic O., Rechberger H. (2022). Product design and recyclability: How statistical entropy can form a bridge between these concepts - A case study of a smartphone. J. Clean. Prod..

[31] Velázquez-Martinez O., Kontomichalou A., Santasalo-Aarnio A., Reuter M., Karttunen A.J., Karppinen M., Serna-Guerrero R. (2020). A recycling process for thermoelectric devices developed with the support of statistical entropy analysis. Resour. Conserv. Recycl..

[32] Velázquez-Martinez O., Van Den Boogart K.G., Lundström M., Santasalo-Aarnio A., Reuter M., Serna-Guerrero R. (2019). Statistical entropy analysis as tool for circular economy: Proof of concept by optimizing a lithium-ion battery waste sieving system. J. Clean. Prod..

[33] Gopan A., Verma P., Yang Z., Axelbaum R.L. (2020). Quantitative analysis of the impact of flue gas recirculation on the efficiency of oxy-coal power plants. Int. J. Greenh. Gas Control.

[34] Luo W., Wang Q., Guo J., Liu Z., Zheng C. (2015). Exergy-based control strategy selection for flue gas recycle in oxy-fuel combustion plant. Fuel.

[35] Seepana S., Jayanti S. (2012). Optimized enriched CO_2_ recycle oxy-fuel combustion for high ash coals. Fuel.

[36] Ignatenko O., van Schaik A., Reuter M.A. (2007). Exergy as a tool for evaluation of the resource efficiency of recycling systems. Miner. Eng..

[37] Meskers C.E.M., Xiao Y., Boom R., Boin U., Reuter M.A. (2007). Evaluation of the recycling of coated magnesium using exergy analysis. Miner. Eng..

[38] Tran H.P., Schaubroeck T., Swart P., Six L., Coonen P., Dewulf J. (2018). Recycling portable alkaline/ZnC batteries for a circular economy: An assessment of natural resource consumption from a life cycle and criticality perspective. Resour. Conserv. Recycl..

[39] Dewulf J., Van der Vorst G., Denturck K., Van Langenhove H., Ghyoot W., Tytgat J., Vandeputte K. (2010). Recycling rechargeable lithium ion batteries: Critical analysis of natural resource savings. Resour. Conserv. Recycl..

[40] Fernandes I., Rudolph M., Hassanzadeh A., Bachmann K., Meskers C., Peuker U., Reuter M. (2021). Thermodynamic analysis of waste heat recovery for cooling systems in hybrid and electric vehicles. Miner. Eng..

[41] Fernandes I.B., Rudolph M., Hassanzadeh A., Bachmann K., Meskers C., Peuker U., Reuter M.A. (2021). The quantification of entropy for multicomponent systems: Application to microwave-assisted comminution. Miner. Eng..

[42] Vakalis S., Patuzzi F., Baratieri M. (2017). Introduction of an energy efficiency tool for small scale biomass gasifiers – A thermodynamic approach. Energy Convers. Manag..

[43] Yang F., Liu Y. (2016). Evaluation and integration of energy utilization in a process system through material flow analysis coupled with exergy flow analysis. Process Saf. Environ. Prot..

[44] Moyaert C., Fishel Y., Van Nueten L., Cencic O., Rechberger H., Billen P., Nimmegeers P. (2022). Using Recyclable Materials Does Not Necessarily Lead to Recyclable Products: A Statistical Entropy-Based Recyclability Assessment of Deli Packaging. ACS Sustain. Chem. Eng..

[45] Singh A., Rorrer N.A., Nicholson S.R., Erickson E., DesVeaux J.S., Avelino A.F., Lamers P., Bhatt A., Zhang Y., Avery G. (2021). Techno-economic, life-cycle, and socioeconomic impact analysis of enzymatic recycling of poly(ethylene terephthalate). Joule.

[46] Sciubba E. (2013). Can an environmental indicator valid both at the local and global scales be derived on a thermodynamic basis?. Ecol. Indic..

[47] Velázquez-Martinez O., Valio J., Santasalo-Aarnio A., Reuter M., Serna-Guerrero R. (2019). A critical review of lithium-ion battery recycling processes from a circular economy perspective. Batteries.

[48] Pinegar H., Smith Y.R. (2019). Recycling of End-of-Life Lithium Ion Batteries, Part I: Commercial Processes. J. Sustain. Metall..

[49] Sonoc A., Jeswiet J., Soo V.K. (2015). Opportunities to improve recycling of automotive lithium ion batteries. Procedia CIRP.

[50] Dunn J.B., Gaines L., Barnes M., Wang M., Sullivan J. (2014).

[51] Metso Outotec HSC Chemistry Software. https://www.mogroup.com/portfolio/hsc-chemistry/.

[52] Bertuol D.A., Toniasso C., Jiménez B.M., Meili L., Dotto G.L., Tanabe E.H., Aguiar M.L. (2015). Application of spouted bed elutriation in the recycling of lithium ion batteries. J. Power Sources.

[53] Hernandez A.G., Cullen J.M. (2016). Unlocking Plant-level Resource Efficiency Options: A Unified Exergy Measure. Procedia CIRP.

[54] Fernandes I.B., Abadías Llamas A., Reuter M.A. (2020). Simulation-Based Exergetic Analysis of NdFeB Permanent Magnet Production to Understand Large Systems. Jom.

[55] Abadías Llamas A., Valero Delgado A., Valero Capilla A., Torres Cuadra C., Hultgren M., Peltomäki M., Roine A., Stelter M., Reuter M.A. (2019). Simulation-based exergy, thermo-economic and environmental footprint analysis of primary copper production. Miner. Eng..

[56] Tanzer J., Rechberger H. (2020). Complex system, simple indicators: Evaluation of circularity and statistical entropy as indicators of sustainability in Austrian nutrient management. Resour. Conserv. Recycl..

[57] Dewulf J., Van Langenhove H., Muys B., Bruers S., Bakshi B.R., Grubb G.F., Paulus D.M., Sciubba E. (2008). Exergy: Its potential and limitations in environmental science and technology. Environ. Sci. Technol..

[58] Vilardi G., Verdone N. (2022). Exergy analysis of municipal solid waste incineration processes: The use of O_2_-enriched air and the oxy-combustion process. Energy.

[59] Huang S.M., Li N.F., Low E., Xiao L., Liang C.H. (2020). Entropy and exergy analysis of a liquid-liquid air-gap hollow fiber membrane contactor. Int. J. Therm. Sci..

[60] Amini S.H., Remmerswaal J.A.M., Castro M.B., Reuter M.A. (2007). Quantifying the quality loss and resource efficiency of recycling by means of exergy analysis. J. Clean. Prod..

[61] Vezzini A., Pistoia G. (2014). Lithium-ion batteries: advances and applications.

[62] Hu Y., Liu F., Li W., Xue F., Li Z., Huang H., Dou C., Zhou X. (2021).

[63] Dai Q., Ren A., Westholm J.O., Duan H., Patel D.J., Lai E.C. (2015). Life cycle analysis of lithium-ion batteries for automotive applications. Genes Dev..

[64] Olivetti E.A., Ceder G., Gaustad G.G., Fu X. (2017). Lithium-Ion Battery Supply Chain Considerations: Analysis of Potential Bottlenecks in Critical Metals. Joule.

[65] Vanderbruggen A., Gugala E., Blannin R., Bachmann K., Serna-Guerrero R., Rudolph M. (2021). Automated mineralogy as a novel approach for the compositional and textural characterization of spent lithium-ion batteries. Miner. Eng..

[66] Latini D., Lagnoni M., Brunazzi E., Mauri R., Nicolella C., DellaPosta P., Tognotti L., Bertei A. (2022). Recycling of Lithium-Ion Batteries: Overview of Existing Processes, Analysis and Performance. Chem. Eng. Trans..

[67] Mossali E., Picone N., Gentilini L., Rodrìguez O., Pérez J.M., Colledani M. (2020). Lithium-ion batteries towards circular economy: A literature review of opportunities and issues of recycling treatments. J. Environ. Manage..

[68] Xu P., Dai Q., Gao H., Liu H., Zhang M., Li M., Chen Y., An K., Meng Y.s., Liu P. (2020). Efficient Direct Recycling of Lithium-Ion Battery Cathodes by Targeted Healing. Joule.

[69] Zhang G., He Y., Wang H., Feng Y., Xie W., Zhu X. (2020). Removal of Organics by Pyrolysis for Enhancing Liberation and Flotation Behavior of Electrode Materials Derived from Spent Lithium-Ion Batteries. ACS Sustain. Chem. Eng..

[70] McLaughlin W., Adams T.S. (1999).

[71] Battaglia G., Berkemeyer L., Cipollina A., Cortina J.L., Fernandez de Labastida M., Lopez Rodriguez J., Winter D. (2022). Recovery of Lithium Carbonate from Dilute Li-Rich Brine via Homogenous and Heterogeneous Precipitation. Ind. Eng. Chem. Res..

[72] Han B., Porvali A., Lundström M., Louhi-Kultanen M. (2018). Lithium Recovery by Precipitation from Impure Solutions – Lithium Ion Battery Waste. Chem. Eng. Technol..

