# Supplementary material for: A multi-dimensional indicator for material and energy circularity: Proof-of-concept of exentropy in Li-ion battery recycling
Source: iScience. 2023 Oct 17;26(11):108237. doi: 10.1016/j.isci.2023.108237 (PMC10637950; doi:10.1016/j.isci.2023.108237)
Supplement: Document S1. Figures S1‒S3 and Tables S1‒S5 [file mmc1.pdf]

iScience, Volume *26*

Supplemental information

A multi-dimensional indicator for material and energy circularity: Proof-of-concept of exentropy in Li-ion battery recycling

Minerva Vierunketo, Anna Klemettinen, Markus A. Reuter, Annukka Santasalo-Aarnio, and Rodrigo Serna-Guerrero

Material flow analysis (MFA) was described in the Introduction of the manuscript. Figure S1 was provided to support the understanding of the concept of MFA, and Figure S1 can also be found in Figure 6 in the main manuscript to support the concept of exentropy.

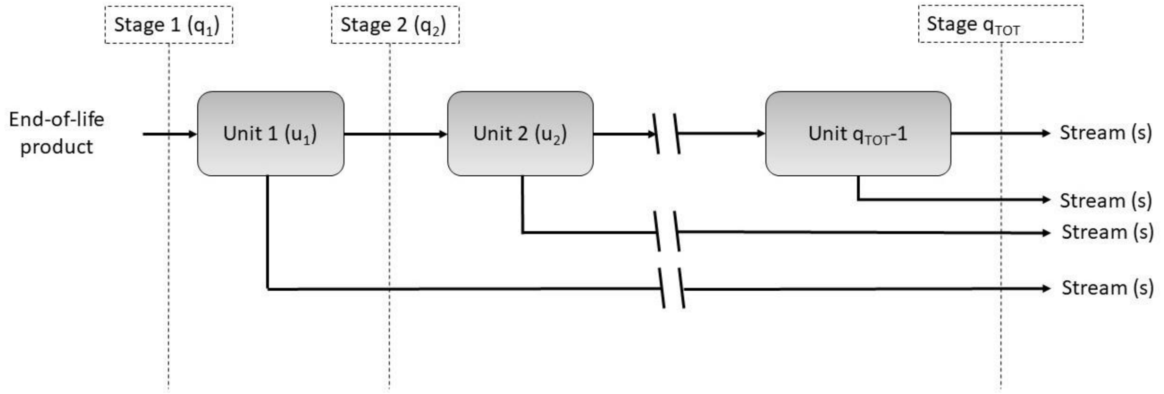

Figure S1: A description of a process according to material flow analysis (MFA), related to STAR Methods, Method details, Statistical entropy analysis.

Tables S1-S4 refer to Figure 2 and Figure 4 in the main manuscript. In Figure 2, the Retriev process for LIB recycling is represented according to the MFA methodology, and in Figure 4, the exergy flows of the Retriev process for LIB recycling using different concentrations of LiOH is represented with Sankey diagrams.

Table S1: Mass flows and exergies of streams during mechanical processing in the Retriev process for LIB recycling with 0.1 M, 1 M, and 2 M LiOH, related to Figure 2.

Stream	Mass flow (kg/h)	Exergy (kW)
S_1	534	2172
S_2	47	238
S_3	13	50
S_4	474	1876
S_5	147	566
S_6	12	49
S_7	315	1261
S_8	86	320
S_9	228	941

Table S2: Mass flows and exergies of streams during hydrometallurgical processing in the Retriev process for LIB recycling with 0.1 M LiOH, related to Figure 2.

Stream	Mass flow (kg/h)	Exergy (kW)
S_{10}	15	2
S_{11}	8200	1059
S_{12}	259	909
S_{13}	7940	151
S_{14}	789	119
S_{15}	7152	205
S_{16}	7263	213
S_{17}	7164	192
S_{18}	99	21
S_{19}	7987	132
S_{20}	111	12

Table S3: Mass flows and exergies of streams during hydrometallurgical processing in the Retriev process for LIB recycling with 1 M LiOH, related to Figure 2.

Stream	Mass flow (kg/h)	Exergy (kW)
S_{10}	15	2
S_{11}	1030	956
S_{12}	259	911
S_{13}	771	45
S_{14}	72	11
S_{15}	699	49
S_{16}	804	56
S_{17}	711	36
S_{18}	93	20
S_{19}	817	28
S_{20}	105	12

Table S4: Mass flows and exergies of streams during hydrometallurgical processing in the Retriev process for LIB recycling with 2 M LiOH, related to Figure 2.

Stream	Mass flow (kg/h)	Exergy (kW)
S_{10}	15	2
S_{11}	631	951
S_{12}	259	913
S_{13}	372	37
S_{14}	33	5
S_{15}	340	38
S_{16}	437	45
S_{17}	351	26
S_{18}	86	19
S_{19}	418	22
S_{20}	97	11

The lithium-ion battery (LIB) composition used in the simulation presented in the main manuscript in the Method details section is presented in Figure S2.

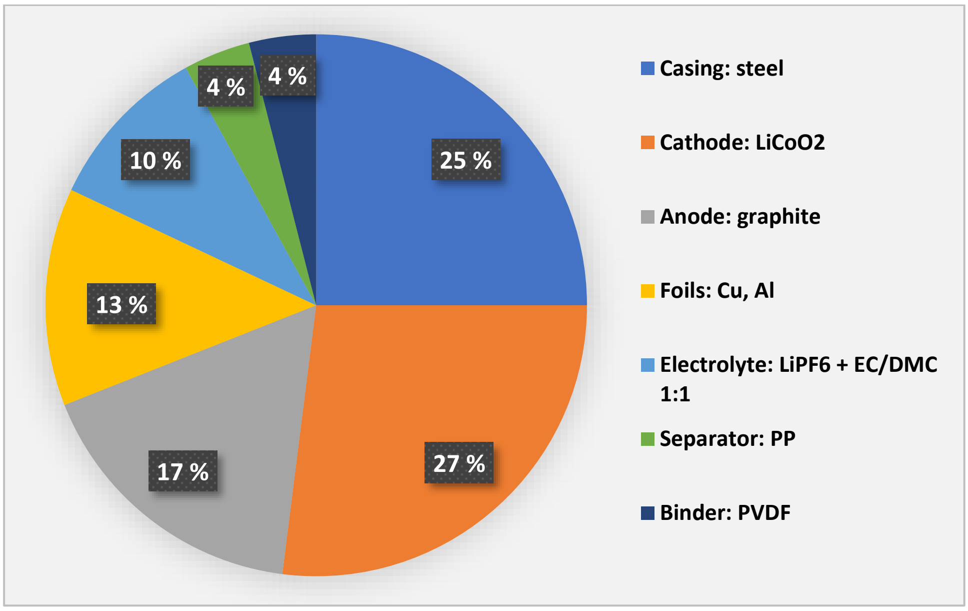

Figure S2: Lithium-ion battery composition used in this study [Data adopted from S1], related to STAR Methods, Method details, Process simulation of battery recycling process.

LIB cells contain various components for their operation, namely cathode and anode active materials, Cu and Al current collectors, electrolyte, and a separator, which are enclosed by a cell casing. Cathode and anode particles are attached on the Al and Cu current collector sheets, respectively, using a binder to secure the electrical conduction and stability of the battery[S2]. The electrodes are placed in an electrolyte to deliver Li-ions between the cathode and anode, and a separator between them that prevents a physical contact and direct electron transfer between the electrodes[S2]. The production of cathode materials, for instance, requires economically valuable metals, as they are usually a lithiated transition-metal oxide or phosphate, e.g., $LiCoO_2$ (LCO), $LiNi_xMn_yCo_z$ (NMO), or $LiFePO_4$ (LFP). Currently, LCO is the most common cathode material due to its prevalence in consumer electronics[S3,S4]. As an anode material, the most common material is graphite, a material currently considered critical by the European Union[S5], although $LiTi_5O_{12}$ can be used in some stationary applications[S1]. The electrolyte is a Li salt (e.g., $LiPF_6$, $LiBF_4$, $LiClO_4$, or $LiSO_2$) dissolved in an organic solvent (e.g., propylene carbonate (PC), ethylene carbonate (EC), or dimethyl carbonate (DMC))[S1]. For the separator, binder, and cell casing porous polymeric material (e.g., polypropylene (PP)), polyvinylidene difluoride (PVDF), and steel or plastics can be used, respectively. For the purposes of this study, we considered a model battery with the composition presented in Figure S2[S1]. Most of the battery mass is comprised by the electrodes, from which the cathode has the largest mass fraction. The casing holds approximately a quarter from the whole battery mass, and components not associated with the electrodes or casing (i.e., electrolyte and separator) occupy only approximately 14% of the whole battery mass.

The LIB recycling process simulation flowsheet is represented in Figure S3. In the main manuscript, the process was described detailed in the Method details section.

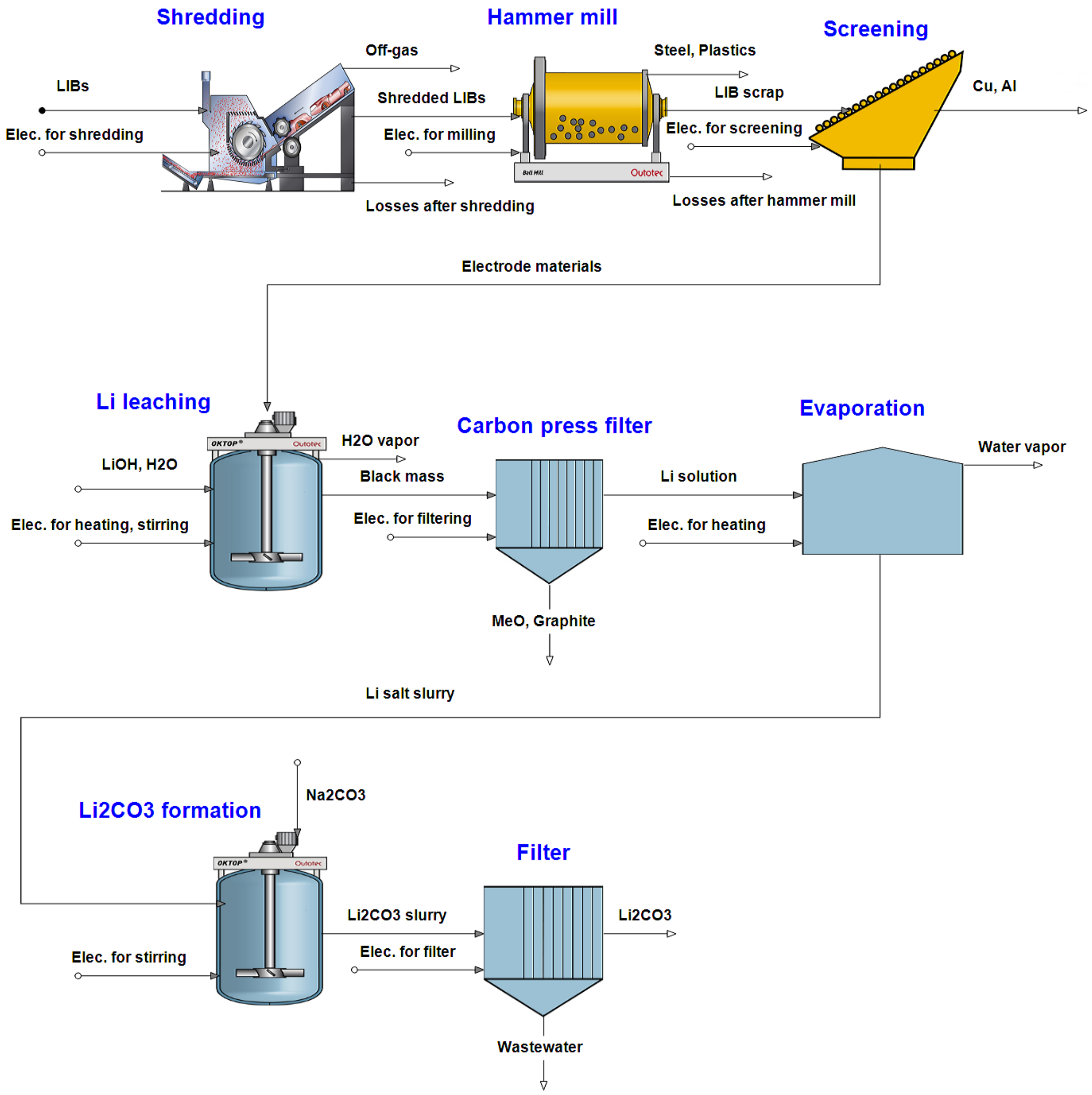

Figure S3: Retriev process simulation flowsheet (Data from S1, S6-S8), related to STAR Methods, Method details, Process simulation of battery recycling process.

The data obtained from the literature for the energy used in different units during the recycling process represented in Figure S3 are listed in Table S5. The energy consumption of the evaporation unit was defined by the simulation software.

Table S5: Energy consumption data for different units from literature used in the simulation, related to STAR Methods, Method details, Process simulation of battery recycling process.

Unit	Value	Reference
Shredding	92 kWh	S6
Hammer mill	60 kWh	S8
Screening	0.95 kWh	S4
Mixing (Li leaching and Li_2CO_3 formation)	2.6 kWh	S4
Carbon press filter	35 kWh	S8
Filter	6.9 kWh	S8

References

S1. Velázquez-Martinez, O., Valio, J., Santasalo-Aarnio, A., Reuter, M., and Serna-Guerrero, R. (2019). A critical review of lithium-ion battery recycling processes from a circular economy perspective. Batteries 5(4), 5—7. 10.3390/batteries5040068.

S2. Bae, H., and Kim, Y. (2021). Technologies of lithium recycling from waste lithium ion batteries: A review. Mater. Adv. 2(10), 3234—3250. 10.1039/d1ma00216c.

S3. Wang, X., Gaustad, G., and Babbitt, C.W. (2016). Targeting high value metals in lithium-ion battery recycling via shredding and size-based separation. Waste Manag. 51, 204—213. 10.1016/j.wasman.2015.10.026.

S4. Wu, F., Li, L., Crandon, L., Cao, Y., Cheng, F., Hicks, A., Zeng, E.Y., and You, J. (2022). Environmental hotspots and greenhouse gas reduction potential for different lithium-ion battery recovery strategies. J. Clean. Prod. 339, 130697. 10.1016/j.jclepro.2022.130697.

S5. European Commission, Directorate-General for Internal Market, Industry, Entrepreneurship and SMEs. Study on the review of the list of critical raw materials: critical raw materials factsheets. Publications Office, 2017, https://data.europa.eu/doi/10.2873/398823.

S6. Pinegar, H., and Smith, Y.R. (2019). Recycling of End-of-Life Lithium Ion Batteries, Part I: Commercial Processes. J. Sustain. Metall. 5(3), 402—416. 10.1007/s40831-019-00235-9.

S7. Sonoc, A., Jeswiet, J., and Soo, V.K. (2015). Opportunities to improve recycling of automotive lithium ion batteries. Procedia CIRP 29, 752—757. 10.1016/j.procir.2015.02.039.

S8. Dunn, J.B., Gaines, L., Barnes, M., Wang, M., and Sullivan, J. (2014). Material and Energy Flows in the Materials Production, Assembly, and End-of-Life Stages of the Automotive Lithium-Ion Battery Life Cycle. United States. 10.2172/1044525.
